# Leukocyte telomere length dynamics in women and men: menopause vs age effects

**DOI:** 10.1093/ije/dyv165

**Published:** 2015-09-18

**Authors:** Christine Dalgård, Athanase Benetos, Simon Verhulst, Carlos Labat, Jeremy D Kark, Kaare Christensen, Masayuki Kimura, Kirsten Ohm Kyvik, Abraham Aviv

**Affiliations:** ^1^Department of Public Health - Environmental Medicine, University of Southern Denmark, Odense, Denmark,; ^2^Département de Médecine Gériatrique, CHU de Nancy, Nancy, France,; ^3^INSERM, U1116, Vandoeuvre-les-Nancy F54000, Nancy, France,; ^4^Université de Lorraine, Nancy, France,; ^5^Groningen Institute for Evolutionary Life Sciences, University of Groningen, Groningen, The Netherlands,; ^6^Hebrew University-Hadassah School of Public Health and Community Medicine, Jerusalem, Israel,; ^7^Danish Twin Registry, University of Southern Denmark, Odense, Denmark,; ^8^Department of Clinical Genetics,; ^9^Department of Clinical Biochemistry and Pharmacology, Odense University Hospital, Odense, Denmark,; ^10^Center of Human Development and Aging, Rutgers, State University of New Jersey, Newark, NJ, USA and; ^11^Institute of Regional Health Services Research, University of Southern Denmark, and Odense Patient Data Explorative Network (OPEN), Odense University Hospital, Odense, Denmark

**Keywords:** Telomeres, women, men, menopause

## Abstract

**Background:** A longer leukocyte telomere length (LTL) in women than men has been attributed to a slow rate of LTL attrition in women, perhaps due to high estrogen exposure during the premenopausal period.

**Methods:** To test this premise we performed a longitudinal study (an average follow-up of 12 years) in a subset of the population-based Danish National Twin Registry. Participants consisted of 405 women, aged 37.5 (range 18.0–64.3) years, and 329 men, aged 38.8 (range 18.0–58.5) years, at baseline examination.

**Results:** Women showed a longer LTL [kb ± standard error(SE)] than men (baseline: 7.01 ± 0.03 vs 6.87 ± 0.04; follow-up: 6.79 ± 0.03 vs 6.65 ± 0.03; both *P* = 0.005). Women displayed deceleration of LTL attrition (bp/years ± SE), as they transitioned from the premenopausal period (20.6 ± 1.0) through the perimenopausal period (16.5 ± 1.3) to the postmenopausal period (15.1 ± 1.7). Age was not associated with LTL attrition in women after statistical control for menopausal status. Men, in contrast, displayed a trend for age-dependent increase in the rate of LTL attrition, which differed significantly from the pattern in women (*P* for interaction = 0.01).

**Conclusions:** Results indicate that the premenopausal period is expressed in a higher rate of LTL attrition than the postmenopausal period. They further suggest that the sex gap in LTL stems from earlier ages—the period of growth and development. The higher rate of LTL attrition in premenopausal women, we propose, might relate to estrogen-mediated increased turnover of erythrocytes, menstrual bleeding or both.

Key Messages

- Women have a longer leukocyte telomere length than men, a finding that was attributed to estrogen stimulating telomerase and thus attenuating age-dependent leukocyte telomere length rate of attrition during the premenopausal period.
- In this longitudinal study we show that the rate of age-dependent leukocyte telomere length attrition is faster during the premenopausal period than postmenopausal period and that regardless of menopausal status leukocyte telomere length is longer in women than men.
- Findings suggest that the sex difference in leukocyte telomere length is largely established prior to adulthood, i.e., during the first two decades of life.

## Introduction

Women typically have a longer leukocyte telomere length (LTL) than men.^1–4^ The underlying causes of this LTL sex difference, though poorly understood, are important because a long LTL is associated with resistance to ageing-related atherosclerotic cardiovascular disease^5^^,^^6^ and with longevity.^7–10^ The prevailing view, derived from cross-sectional studies,^11^ is that a slower rate of age-dependent LTL attrition in women explains the LTL sex gap. However, two longitudinal studies reported conflicting findings. Chen *et al.*^12^ showed that the rate of LTL attrition was slightly slower in premenopausal women than men (by 4.5 bp/year, *P* = 0.04), whereas a subsequent study by Kark *et al.*^13^ suggested that the rate of LTL attrition was slightly more rapid in premenopausal women (by 2.5 bp/year, *P* = 0.081). Thus, the effect of sex and menopausal status on LTL attrition warrants scrutiny, and its relation to the LTL sex gap remains unresolved.

A slower LTL attrition in women, if true, might stem from the effect of estrogen on telomerase, the reverse transcriptase that adds telomere repeats to the ends of chromosomes.^14^ As an estrogen-response element is present in the promoter of the catalytic subunit of telomerase, estrogen can potentially stimulate telomerase^15^ to attenuate the rate of age-dependent LTL shortening in premenopausal women and thereby explain, at least in part, the LTL sex gap. Although telomerase activity is repressed in somatic tissues during extra-uterine life, it is not totally absent from highly proliferative tissues such as the haematopoietic system and skin.^16^ Accordingly, we tested the dual proposition that the rate of LTL attrition differs by menopausal status and that a putatively slower rate of LTL attrition during the premenopausal period partly explains the longer LTL in women than in men. Our findings support the first proposition, but cast doubt on the latter one.

## Methods

### Subjects

Healthy subjects were originally recruited through the population-based Danish National Danish Twin Registry^17^^,^^18^ to participate in the GEMINAKAR Study of metabolic disorders and cardiovascular risk factors, performed in Odense and Copenhagen, Denmark. For this specific investigation, the sample consisted of 338 same-sex (SS) twin pairs (i.e. 676 twins) and 72 opposite-sex (OS) twin pairs (i.e. 144 twins). In total, 75 twin SS and 11 twins OS were excluded due to the absence of LTL measurements either at the first or at the second visit. Thus, all subjects included in this analysis had data on LTL measurements, BMI, smoking status and menopause status for women in both baseline and follow-up visits. We used the GEMINAKAR Study not for its twin structure but because of the availability of longitudinal leukocyte DNA samples for LTL measurements and wealth of phenotypic data that included menopausal status. At baseline, participants were without clinical evidence of diabetes or cardiovascular disease. Participants underwent comprehensive anthropometric examination and provided relevant information and blood samples approximately 12 years apart. Women with pathological menopause, namely women subjected to oophorectomy and/or hysterectomy (*n* = 32) and those reporting menopause (absence of menses for at least 1 year) at age < 42 years (*n* = 22) were included. The mean age of menopause in this cohort was 47.4 ± 6.0 years. We stratified women participants into three categories: postmenopausal women, i.e. women who had menopause before the baseline examination; perimenopausal women, i.e. women who experienced menopause (i.e. the absence of menses for 1 year) between baseline and follow-up examinations; and premenopausal women, i.e. women who did not reach menopausal age at follow-up examination. The study was approved by the Regional Scientific Ethical Committees for Southern Denmark (S-20090065) and the Danish Data Protection Agency (2013-41-1556). All participants provided written informed consent.

### Leukocyte telomere length (LTL) measurements

We performed measurement of the terminal restriction fragments by Southern blots as previously described.^19^ Measurements were performed in duplicate on different gels and analyses are based on the averages of these two measurements. The inter-assay coefficient of variation for the duplicate measures was 1.3%.

### Statistical analysis

In previous work on the same cohort,^20^ we showed that among SS twins, as reported for singletons in the general population, females had a longer LTL than males. In contrast, LTL of females from OS twins was indistinguishable from that of males from either OS or SS twins, and was shorter than LTL of females from SS twins. In addition, there was no evidence in the previous work that LTL or LTL attrition differed between mono- and dizygotic twins. For these reasons, in our analyses we controlled for twin type (OS vs SS), interacting with sex and age, but ignored zygosity throughout (Tables 1 and 2). Data were analysed with general linear mixed models, including twin identity as a random effect because LTL measurements of co-twins are not statistically independent. Including body mass index (BMI) or smoking status in the model did not affect the results (data not shown), and for simplicity we therefore excluded these factors from the models. Because of the complexity of the longitudinal analyses, we analysed the sexes separately and subsequently pooled the data sets to test interactions with sex for the pertinent results. Individuals were examined twice. We partitioned age at examination into two variables: the average age at examination and the deviation in age of each individual for each examination from the average age for that individual. For example, for an individual whose LTL was measured at ages 20 (baseline examination) and 30 (follow-up examination), the average age is 25 for both examinations, whereas the age difference (delta) is −5 and +5 for the baseline examination and follow-up examination, respectively. This procedure yields an unbiased estimate of the within-individual effect of age on LTL.

**Table 1.** Longitudinal analysis of LTL (kb) in relation to age and twin sex composition (opposite-sex vs same-sex)

A. Women^a^	Estimate (95% CI)	F (df)	*P*
Intercept	7.995 (7.695, 8.295)	−	<0.0001
Average age	−0.0243 (−0.0310, −0.0176)	49.72 (1, 276.8)	<0.0001
Delta (Δ) age	−0.0262 (−0.0327, −0.0197)	663.31 (1, 401.2)	<0.001
OS	−0.230 (−0.391, −0.070)	7.83 (1, 297.6)	0.0055
Δ Age * OS	0.0065 (0.0024, 0.0106)	10.03 (1, 401.2)	0.0017
Δ Age * average age	0.00016 (0.000023, 0.000297)	4.41 (1, 401.2)	0.036

B. Men^b^	Estimate (95% CI)	F (df)	*P*

Intercept	7.611 (7.270, 7.952)		<0.0001
Average age	−0.0191 (−0.0262, −0.0120)	28.69 (1, 259.1)	<0.0001
Delta (Δ) age	−0.0142 (−0.0213, −0.0071)	15.82 (1, 326.7)	<0.0001
OS	−0.042 (−0.206, 0.122)	0.253 (1, 233.6)	0.62
Δ Age * OS	0.0067 (0.0030, 0.0104)	11.81 (1, 328)	0.0007
Δ Age * average age	−0.00012 (−0.00028, 0.000037)	2.51 (1, 326.7)	0.11

CI, confidence interval; OS, opposite sex; SS, same sex.

OS was coded 1 for OS twins and 0 for same-sex (SS) twins.

Twin identity and individual identity, nested in twin identity, were included as random effects.

^a^For women, *n* = 841, R^2 ^= 0.983.

^b^For men, *n* = 693, R^2 ^= 0.984.

**Table 2.** Longitudinal analysis of women^a^ LTL (kb) in relation to menopausal stage, age and twin sex composition (opposite sex vs same sex)

	Estimate (95% CI)	F (df)	*P*
Intercept			
Average age	–	28.86 (1, 396.4)	<0.0001
Delta (Δ) age	–	484.13 (1, 399.3)	<0.0001
Opposite sex (OS)	–	7.71 (1, 296.9)	0.0058
Δ Age * OS	–	9.54 (1, 399.3)	0.0021
Menopause stage	–	0.0257 (2, 385.5)	0.97
Δ Age * menopause stage	–	5.42 (2, 399.3)	0.0048
Premenopause slope	−0.0206 (−0.0226, −0.0186)	–	–
Perimenopause slope	−0.0165 (−0.0191, −0.0140)	–	–
Postmenopause slope	−0.0151 (−0.0184, −0.0118)	–	–

Menopause is a fixed effect factor coding for pre-, peri- and postmenopausal.

^a^*n* = 839; R^2 ^= 0.984.

Analyses were performed using JMP 9.0.1.

## Results

**Figure 1.**
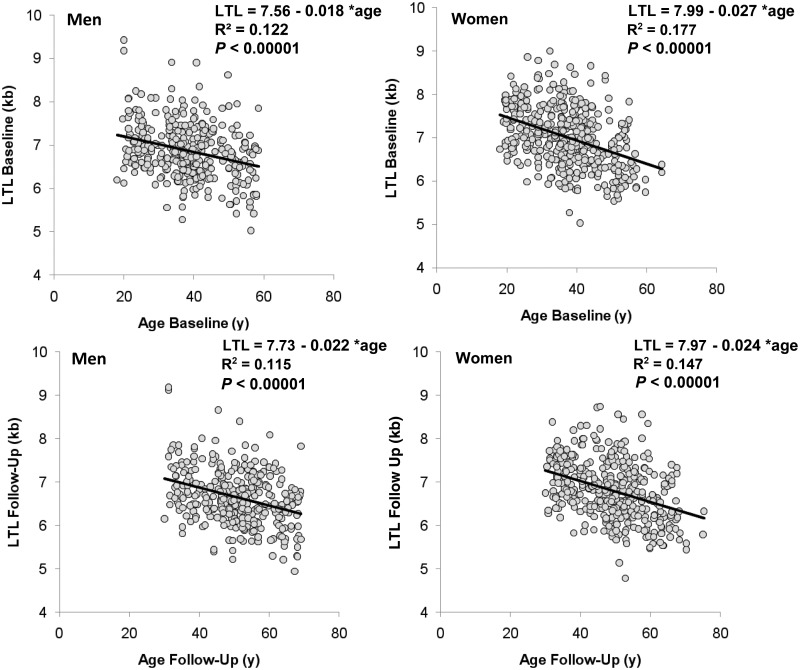
Leukocyte telomere length vs age in men and women; y, years. Data of baseline examination are presented in the upper panels and data of the follow-up examination are presented in the lower panels. Parameters of the linear regressions are displayed in each of the panels (from model including twin identity as random effect; R^2^ in the graphs refer to increase in R^2^ due to the age effect, given the twin identity effect).

General characteristics of the subjects are displayed in Table S1 (available as Supplementary data at *IJE* online). Women showed a longer LTL (kb; mean ± SE), than men both at baseline (7.01 ± 0.03 vs 6.79 ± 0.03; *P* = 0.005) and at follow-up (6.87 ± 0.04 vs 6.65 ± 0.03; *P* = 0.005). Based on cross-sectional analysis, both women and men displayed inverse relations between LTL and age at the baseline and follow-up examinations (Figure 1).

Applying the model described under Methods, we first examined the within-individual effect of age on LTL attrition by testing the interaction between the deviation in age of each individual at each examination from the average age for that individual (delta age) and the mean age. We found an interaction between delta age and mean age for women, and there was a trend for men (Table 1). More importantly, the signs of the interactions were opposite, such that LTL attrition decreased with average age in women and it increased with average age in men (three-way interaction Sex * Average age * Delta age: F1, 728.1 = 6.64, *P* = 0.01). Notably, despite the sex difference in the dependence of LTL attrition on age, when ignoring average age at sampling there was no difference between the sexes in the rate of LTL attrition (interaction sex * delta age: F_1,730_ = 0.05, *P* = 0.8; men: 18.4 ± 0.8 (bp/year; mean ± SE), women: 18.5 ± 0.7), indicating that the overt sex differences evident during early and later life cancelled each other out in this sample.

**Figure 2.**
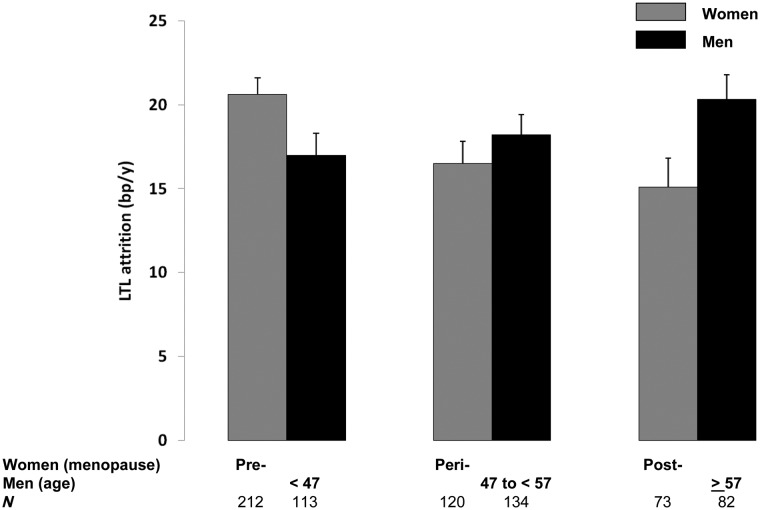
Comparisons of leukocyte telomere length attrition between women and men. Comparisons between women and men are based on the menopausal status of the women and the age of the men at the follow-up examination, which falls in the range of the age of the women categorized for their specific status; y, years. Data are presented as mean ± SE. For details, see text and Tables 2 and 3.

Next we tested whether the slowing down of LTL attrition with age in women could be attributed to menopausal status as opposed to age per se. When replacing the average age* delta age interaction in the model in Table 1A with both menopausal status and its interaction with delta age (Table 2), we detected this interaction with the rate of LTL attrition decreasing from pre-, to peri-, to postmenopausal state (Figure 2). In this analysis (Table 2), menopausal status is confounded by age. To test whether menopausal status may explain the association of average age with LTL attrition, we added the interaction between average age and delta age to the model in Table 2. In this expanded model (not shown), there was no evidence for interaction between average age and delta age (F_1,398.3_ = 0.14, *P* = 0.7), whereas the interaction between menopausal status and delta age was attenuated but persisted (F_2,398.3_ = 3.25, *P* < 0.04). Thus we conclude that menopausal status is associated with LTL attrition independently of age. Notably, a subset of the women had experienced pathological menopause (see Methods), but results held when these women were excluded (Figure S1, available as Supplementary data at *IJE* online).

We tested whether hormone replacement therapy was associated with LTL, since 17.7% of women responded affirmatively to the question whether or not they had ever used hormone replacement therapy. We tested this both by itself and in interaction with delta age in models presented in Table 1A and Table 2. Neither use of hormone replacement therapy nor its interaction with delta age yielded an increase in the explained variance (all *P* > 0.3) or substantially changed the other estimates. Similarly, we tested whether the use of oral contraceptives at baseline was associated with LTL and/or LTL attrition. Reported oral contraceptive intake was 20.9% for premenopausal women, and 7.5% for perimenopausal women. To test for possible effects of oral contraceptive use, we selected premenopausal women and tested the effect by itself and in interaction with delta age by adding these terms to the model presented in Table 1A. Neither use of oral contraceptives nor its interaction with delta age yielded a significant increase in the explained variance (both *P* > 0.4).

Based on these findings, we repeated the sex comparison in Table 2 for different menopausal stages. To this end, the data for men were assigned to one of three age groups corresponding to the ages of the menopausal stages in women (see Subjects and Methods for details). The association of menopausal stage with telomere attrition differed between the sexes (three-way interaction menopause stage * sex * delta age: F_2,724.9_ = 5.05, *P* = 0.007). The comparisons between the sexes for the different menopausal stages showed that in younger individuals (premenopausal) LTL attrition was higher in women than in men (F_1,322.8_ = 2.22, *P* = 0.10), in middle age (perimenopausal) there was no difference and in the older individuals (postmenopausal) attrition was higher in men than in women (F_1,152_ = 6.11, *P* < 0.02).

## Discussion

Women have a longer LTL than men,^1–4^ as confirmed in the present study. The sex difference in LTL might stem from sex-related differences in LTL dynamics before adulthood or it could arise later if women have a lower rate of LTL attrition. We tested the latter hypothesis through longitudinal evaluations of the rates of LTL attrition in the two sexes. Our findings provide an unexpected portrayal of LTL dynamics in adults.

We found that LTL attrition decreased with age in women, whereas there was a trend for an increase in LTL attrition with age in men. This implies that the outcome of a comparison of LTL attrition between the sexes will depend on the age of the sample. The findings of Kark *et al.*^13^ based on a longitudinal study of 13 years and a mean age of 30 years at baseline, are consistent with this conclusion. In that study, women who were almost entirely in the premenopausal period exhibited slightly faster rates of LTL attrition than men of the same age. A more recent longitudinal study of 6.6 years’ duration, using a qPCR-based method to measure LTL, observed a sex x age interaction in LTL attrition^21^ with faster LTL attrition in men than in women. Menopause was not taken into consideration. As age at baseline examination in that study was 48 years, which is equivalent to the mean menopausal age of 47.4 years in the present study, its findings are also consistent with our conclusion.

More detailed analysis showed that among women the premenopausal period was marked by a faster rate of LTL attrition than the postmenopausal period. The LTL attrition rate during perimenopause was intermediate between the premenopausal and postmenopausal LTL attrition rates, but was closer to the postmenopausal rate than to the premenopausal rate, suggesting that LTL attrition had already slowed down before menopause.

Collectively, this body of data suggests that the sex difference in LTL attrition rate, across the age span studied, stems from the impact of menopausal status on LTL attrition in women. However, telomerase stimulation by endogenous estrogen and its anti-oxidative effect through mitochondrial action^22^ during the premenopausal period are unlikely to explain the longer LTL in women than men, since the rate of LTL attrition during the premenopausal period was in fact faster than that during the postmenopausal period. We found no effect of exogenous estrogen intake on the rate of LTL attrition. However, since only a small subset of women were on oral contraceptives or hormone replacement therapy, power to detect the effect of exogenous estrogen on LTL attrition was limited.

LTL attrition largely reflects the asymmetrical replication of haematopoietic stem cells (HSC).^23^^,^^24^ As the ratio between erythrocytes and leukocytes in the blood cell mass is ∼ 800 to 1^25^, the vast size of the erythrocyte pool is the principal driving force of HSC asymmetrical replication. In that light a recent work shows that, in mice, estrogen enhances erythrocyte turnover by stimulating HSC asymmetrical replication in concert with increasing erythrocyte destruction.^26^ The outcome of this phenomenon, if extended to humans, would be a faster LTL attrition during the premenopausal period, as found in our study.

Menstrual bleeding might be another potential explanation for the faster LTL attrition in premenopausal women. The average blood loss per menstrual period is estimated at 40 ml, although with a wide range.^27^ This amounts to ∼ 500 ml/year, which may account at least in part for the findings of the faster rate of LTL attrition in premenopausal vs postmenopausal women. Diminished menstruation as women approach menopause might also explain the finding that LTL attrition during perimenopause was closer to the postmenopausal rate than to the premenopausal rate.

Our longitudinal evaluation overcomes the serious shortcomings of assessing rates of LTL attrition based on cross-sectional studies, which consist of individuals of different ages whose LTL is highly variable for a given age (∼3 kb, Figure 1). Whereas longitudinal evaluation of LTL attrition provides direct observations of the individual’s rate of attrition, inference from these observations is constrained by the error of telomere length measurements in relation to the duration of the follow-up.^28^ We would like to emphasize, however, that although confidence in our findings is reasonably high (given the average 12-year duration of the follow-up and the low measurement error of the Southern blots of LTL), the findings are inconsistent with the previous prevailing view; therefore, they invite replication in future longitudinal studies.

In addition, for the following reasons the potential explanations we offer for our findings should be guarded. First, we propose that the higher rate of LTL attrition in premenopausal women might stem from a higher turnover of HSCs based on data derived from mice; whether this finding extends to humans remains to be verified. Second, with regard to the potential impact of menstrual blood loss on LTL attrition, we have no detailed information in this study about the extent of menstrual bleeding in the women participants, nor how it compares in scale with total erythrocyte turnover. Third, pregnancy entails an increase in the maternal blood volume which, in principle, might impact on the rate of LTL attrition during the reproductive years. However, in this study no data were available about reproduction during the follow-up period. Finally, our findings are based on data in healthy twins, free of diabetes and cardiovascular disease at the baseline examination, and both co-twins had to survive to the follow-up examination. Thus, our healthy cohort of twins may not be representative of the general population, which might explain, for instance, the low rate of age-dependent LTL attrition we report in this study.

Based on direct and indirect assessments of LTL dynamics during early life,^28–31^ previous studies have reached the conclusion that the longer LTL in women than men is the outcome of a difference established before adulthood. In a small study consisting of three ethnic groups (Whites of European ancestry, African Americans and Hispanics), LTL in newborn girls was found to be longer by 57 bp than that in boys.^32^ However, this difference was not statistically significant. In light of our findings, large-scale studies are warranted to establish whether the sex difference in LTL stems from a longer LTL in newborn girls than boys, a slower LTL attrition during childhood in girls than boys, or both.

Having more clarity as to the origin of the sex gap in LTL might lead to mechanistic understanding of factors that drive LTL dynamics. This will be highly relevant to public health, given that short LTL entails an increased risk for atherosclerotic heart diseases in adults^5^ and diminished longevity.^7–10^ Whether telomeres play a causal role in atherosclerosis and longevity remains unresolved, but a growing body of evidence points to such a possibility.

## Supplementary data

Supplementary data ate available at *IJE* online.

## Funding

This work was supported by NIH grants AG030678 and HD071180; the Danish Council for Independent Research—Medical Sciences; the INTERREG 4 A programme Southern Denmark-Schleswig-K.E.R.N. supported by the European Regional Development Fund; and the A.P. Møller Foundation for the Advancement of Medical Science.

**Conflict of interest:** None declared.

## Supplementary Material

Supplementary Data
