# Supplementary material for: Leukocyte telomere length dynamics in women and men: menopause vs age effects
Source: Int J Epidemiol. 2015 Sep 18;44(5):1688–95. doi: 10.1093/ije/dyv165 (PMC4681111; doi:10.1093/ije/dyv165)
Supplement: Supplementary Data [file supp_44_5_1688__index.html]

Leukocyte telomere length dynamics in women and men: menopause vs age effects — Supplementary Data 

# Leukocyte telomere length dynamics in women and men: menopause vs age effects

## Supplementary Data

files

- Supplementary Data - docx file
