# Supplementary material for: Leukocyte telomere length dynamics in women and men: menopause vs age effects
Source: Int J Epidemiol. 2015 Sep 18;44(5):1688–95. doi: 10.1093/ije/dyv165 (PMC4681111; doi:10.1093/ije/dyv165)
Supplement: Supplementary Data [file supp_dyv165_Supp_Data.docx]

**Supplementary Data**

|  | | | | |
| --- | --- | --- | --- | --- |
| **Table S1 . Subject Characteristics** | | | | |
| **Parameter** | **All** | **Men** | **Women** | **p** |
|  | **(n = 734)** | **(n = 329)** | **(n = 405)** | **(sex)** |
| **Baseline examination** |  |  |  |  |
| Age, years | 38.1 (9.9) | 38.8 (9.9) | 37.5 (9.9) | 0.08 |
| BMI, kg/m2 | 24.4 (3.4) | 25.1 (3.2) | 23.9 (3.5) | < 0.00001 |
| Smokers, % | 30.7% | 31.3% | 30.1% | 0.73 |
| LTL_B_, kb | 6.95 (0.67) | 6.87 (0.65) | 7.01 (0.68) | 0.005 |
|  |  |  |  |  |
| **Follow-up examination** |  |  |  |  |
| Age, years | 50.1 (9.9) | 50.7 (9.8) | 49.5 (9.9) | 0.12 |
| BMI, kg/m2 | 25.7 (4.1) | 26.3 (3.7) | 25.2 (4.4) | 0.0002 |
| Smokers, % | 20.7% | 19.7% | 21.5% | 0.54 |
| LTL_FU_, kb | 6.72 (0.67) | 6.65 (0.62) | 6.79 (0.65) | 0.005 |
| Data are presented as mean with SD | | | | |

**
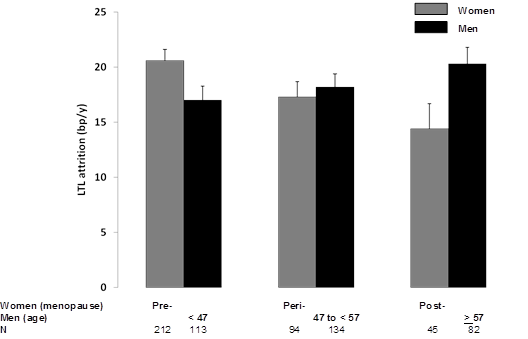
**

**Figure S1. Comparisons of leukocyte telomere length attrition between women and men** Data are as in Figure 2 in the paper, but for women the estimates were calculated after omitting women with pathological menopause from the data set (n=57 women excluded, leaving n=387 women, measured twice). The interaction between menopause state and delta age is statistically significant in this restricted data set (women only: F_2,346.4_ = 4.11, P=0.017), as is the three-way interaction between menopause state, delta age and sex (F_2,672.3_ = 4.61, P=0.01). Comparisons of the leukocyte telomere length attrition rates between women and men are based on the menopausal status of the women and the age of the men, which falls in the range of the age of the women categorized for their specific status. Data for men is as per Figure 2. Data are presented as mean ± SE.
